# Clinical and Laboratory Features of Scrub Typhus among Adult Patients with Acute Febrile Illness in Tak and Nakhon Phanom Provinces, Thailand, 2017–2020

**DOI:** 10.4269/ajtmh.25-0551

**Published:** 2026-07-14

**Authors:** Adelaide F. Green, Betsy Cadwell, Emily Bloss, Nuttagarn Chuenchom, Supphachoke Khemla, Sumonmal Uttayamakul, Pongpun Sawatwong, Ornuma Sangwichian, Duangkamon Siludjai, Phanthaneeya Teepruksa, Beth Skaggs, Cecilia Y. Kato, Christopher J. Gregory, John R. MacArthur, James D. Heffelfinger, Stephanie J. Salyer, Saithip Bhengsri

**Affiliations:** ^1^ Epidemic Intelligence Service, Centers for Disease Control and Prevention, Atlanta, Georgia, USA;; ^2^ Division of Global Health Protection, Global Health Center, Centers for Disease Control and Prevention, Atlanta, Georgia, USA;; ^3^ Division of Global Health Protection, Thailand Ministry of Public Health-United States of America Centers for Disease Control and Prevention Collaboration, Nonthaburi, Thailand;; ^4^ Mae Sot Hospital, Tak, Thailand;; ^5^ Nakhon Phanom Hospital, Nakhon Phanom, Thailand;; ^6^ Department of Disease Control, Ministry of Public Health, Bamrasnaradura Infectious Diseases Institute, Nonthaburi, Thailand;; ^7^ Division of Vector-Borne Diseases, National Center for Emerging and Zoonotic Infectious Diseases, Centers for Disease Control and Prevention, Atlanta, Georgia, USA

## Abstract

Scrub typhus (ST), caused by *Orientia tsutsugamushi*, is an underdiagnosed cause of acute febrile illness in Southeast Asia. Acute febrile illness surveillance data from febrile patients presenting to health facilities in two Thailand border provinces (Tak [north] and Nakhon Phanom [northeast]) were analyzed to estimate ST prevalence and describe clinical and epidemiological characteristics and risk factors. Scrub typhus cases were classified as confirmed (polymerase chain reaction-positive), probable (acute-to-convalescent rise in ELISA IgM/IgG plus ≥2 clinical features), or suspect (positive acute and convalescent sera results for anti-IgM/IgG or for patients with only one sera sample, positive acute or convalescent sera results for anti-IgM/IgG and ≥2 of these clinical features). All others were considered non-cases. The 75 ST cases were classified as confirmed (39) or probable (36). Scrub typhus prevalence was higher in Tak (6.9%; 95% CI: 4.8–9.4) than in Nakhon Phanom (1.3%, 95% CI: 0.6–2.3). Common symptoms included headache, fatigue, myalgia, and chills. Discharge diagnosis matched ST case designation 15% of the time. Approximately one-quarter (*n* = 18) of patients were coinfected with another bacterium (15, 20%) or virus (3, 4%). Appropriate antibiotics were administered to 56% (*n* = 9) of the 21% (*n* = 16) of patients who received antibiotics before study specimen collections. Participants in Tak were 4.2 (95% CI: 2.2–8.2) or 4.5 (95% CI: 2.4–8.5) times as likely to test positive for ST if they visited the forest or contacted farm animals, respectively, within the past 30 days. Findings confirm the persistent burden of ST along Thailand’s borders. Targeted actions are needed to strengthen clinical management, access to diagnostics, and community-based prevention.

## INTRODUCTION

Acute febrile illness (AFI) is characterized by a rapid onset of fever without localized symptoms. Acute febrile illness severity ranges from a mild, self-limiting, febrile illness to a life-threatening disease.^1^ With nondescript symptoms and multiple causes, the diagnosis and management of AFI is particularly difficult. Infectious causes of AFI are numerous and broadly include bacterial, viral, fungal, and parasitic pathogens.^1^^,^^2^ Globally, surveillance platforms have been put in place to help determine the predominant causes of AFI and inform prevention, control, and clinical management efforts. Neglected tropical diseases like scrub typhus^3^ have been found to be one of the leading bacterial causes of AFI in Asia and, more specifically, Thailand.^4^

Scrub typhus (ST) is a vector-borne disease caused by *Orientia tsutsugamushi* (*O. tsutsugamushi*) of the family *Rickettsiaceae,* which includes the genera *Rickettsia* and *Orientia.*^5^^,^^6^ Scrub typhus is transmitted to humans by larval mites, which usually feed on small mammals, such as rodents.^7^ Clinical manifestations of ST include fever, myalgia, headache, and cough, which are almost indistinguishable from other causes of AFI. A characteristic eschar at the ectoparasite bite site is more specific as a diagnostic clue; however, there is wide variation in its occurrence, appearing in anywhere between 20% and 90% of cases.^8^^–^^10^ Scrub typhus infection has also been associated with acute kidney injury, acute respiratory distress syndrome, multiorgan dysfunction, and central nervous system infections.^11^^–^^13^ Serological tests have been the gold standard for the diagnosis of ST; however, these require technical expertise and infrastructure, making their application in clinical settings less practical. Antibiotics considered effective against ST include doxycycline, chloramphenicol, rifampin, and azithromycin.^14^ Although ST is readily treatable with doxycycline, the nonspecific AFI clinical features and limited availability of diagnostics in clinical settings can lead to underdiagnosis and inappropriate treatment.^9^^,^^15^^,^^16^ Untreated ST in Asia has been found to have a median mortality of 6% (range: 0%–70%).^17^

To improve early diagnosis, appropriate treatment, and awareness of rickettsioses in patients with AFI, clinical and laboratory data have previously been used to develop predictor-based clinical decision algorithms that differentiate rickettsial infections from other infectious causes of AFI.^9^^,^^18^ Several previous etiological studies in Thailand have provided epidemiological evidence of ST contributing to AFI. However, there have been limited studies conducted using integrated clinical and laboratory AFI data to diagnose ST in border areas, where there is frequent cross-border movement, and hospitals often have limited resources.^19^^–^^22^ Evidence of cross-border movement of ST is suggested by closely related isolates previously found in the Laos–Thai border areas.^23^

As part of a large-scale, enhanced laboratory-based surveillance of AFI in the border regions of Thailand, the prevalence of ST among adult patients hospitalized with AFI in Tak and Nakhon Phanom (NP) Provinces between 2017 and 2020 was estimated. The clinical features, exposure history, accuracy of discharge diagnosis, and antibiotic therapy in the 72 hours before hemoculture were further described for participants retrospectively determined to be a confirmed or probable ST case.

## MATERIALS AND METHODS

### Design.

The data analyzed are from a prospective observational cohort study conducted in Tak and NP Provinces from April 2017 to May 2020.

### Setting.

Tak Province is located in lower Northern Thailand along the Thailand–Myanmar border, with a population of 665,620 (2019) in its nine districts. Nakhon Phanom Province is located in Northeastern Thailand along the Thailand–Laos border, with a population of 719,136 (2019) in its 12 districts. The present study was conducted in the same five hospitals of Tak and seven hospitals of NP as described in Wodniak et al. (2024).^24^ Both provinces have a tropical climate with wet and dry seasons.

### Population.

Enrollment included individuals aged 2 to 80 years who were admitted to participating hospitals with a temperature ≥38°C (oral, tympanic membrane, or rectal temperature) or a history of fever lasting ≤7 days. Patients were excluded if they returned for continued treatment of a known cause of fever within the previous 30 days, were admitted to any hospital within the previous 14 days prior, were not legal residents of Thailand, or could not read or understand Thai, Lao, Burmese, or Karen languages. Patients were categorized as having AFI if they had localizing symptoms (≥3 diarrheal events in the previous 24 hours or ≥2 respiratory symptoms), or as having acute undifferentiated febrile illness (AUFI) without localizing symptoms.

### Data and specimen collection.

Specimens were collected for laboratory testing from all AUFI and a random sample of 20% of patients categorized as AFI. Informed consent was obtained, and data collection occurred as described in Wodniak et al. (2024).^24^ All clinical assessments conducted during admission and hospitalization were performed by healthcare professionals as part of routine clinical management. Patient interviews were conducted by trained project staff to collect demographic information, clinical manifestations at admission, and exposure history potentially related to the current illness. Medical record reviews were conducted to extract pertinent information during the hospitalization period using a standardized data collection form, including available routine laboratory results, discharge diagnoses, and comorbidities. In the present analysis, demographic, exposure history, clinical manifestation, discharge diagnosis, antibiotic use before hemoculture sample collection, and laboratory diagnostic result variables were used. Notably, hemoculture sample collection was performed as a part of the AFI study from which the study data subset came. Hemoculture itself is not relevant to ST diagnostics in the present study. The variable for antibiotic use within the 72 hours before hemoculture sample collection is the only study variable available related to antibiotic use and is used to compare empiric antibiotic use with retrospectively classified confirmed or probable ST diagnoses.

### Laboratory assays.

At the respective hospitals, blood and urine specimens were collected from patients upon enrollment (acute specimens), and convalescent blood specimens were collected ∼30 days (21–35 days) after the acute specimen.

#### Molecular assays.

Total nucleic acids (TNAs) were extracted from whole-blood samples using the MagNA Pure Compact automatic extraction machine (Roche Diagnostics, Indianapolis, IN, USA). Singleplex polymerase chain reaction (PCR) was used to separately identify the presence of *O. tsutsugamushi* in blood specimens. Multiplex real-time PCR was performed for each TNA from ethylenediaminetetraacetic acid (EDTA) whole blood by using the QuantStudio 7 Flex Real-Time PCR System (Thermo Fisher Scientific, Waltham, MA, USA). A 384-well TaqMan Array Card (TAC) customized by the U.S. Centers for Disease Control and Prevention (CDC) was used to detect 27 pathogens, including 15 bacteria (*Bartonella* spp., *Brucella* spp., *Burkholderia pseudomallei* (*B. pseudomallei*)*, Coxiella burnetii, Escherichia coli* (*E. coli*)*/Shigella, Streptococcus pyogenes, Leptospira* spp. pathogenic strain*, O. tsutsugamushi, Rickettsia* spp., *Salmonella* spp., *Salmonella Paratyphi A, Salmonella Typhi, Streptococcus pneumonia, Streptococcus suis,* and *Yersinia* spp.), 10 viruses (chikungunya virus, dengue virus [pan], dengue virus serotypes 1 to 4, hepatitis E virus, Japanese encephalitis virus, Nipah virus, and Zika virus), and two parasites (*Plasmodium falciparum* and *Plasmodium vivax*). Internal Positive Control and RNase P Control were also included in the TAC. Fifty microliters of each nucleic acid sample, Positive Control, and No Template Control were thoroughly mixed with 50 *µ*L of qScript pre-mix (Quantabio, Beverly, MA, USA) and added into each fill reservoir of the TAC. After two rounds of centrifugation at 1,200 rpm (relative centrifugal force of 314 × g) for 1 minute each using a Heraeus Megafuge 40 centrifuge with a 195-mm swing-bucket rotor (cat. no. 75003180; Thermo Fisher Scientific), the TAC cards were sealed, and the fill reservoirs were removed. The TAC was loaded into the instrument (PCR conditions: 45°C for 10 minutes and 94°C for 10 minutes, 45 cycles of 94°C for 30 seconds and 60°C for 1 minute). The results were analyzed by using QuantStudio Real-Time PCR software version 1.2 (Thermo Fisher). All specimens for molecular assays were transferred to the Bamrasnaradura Infectious Disease Institute, Department of Disease Control, Ministry of Public Health in Nonthaburi, Thailand. The molecular assay results were reported to clinicians monthly.

#### ELISA testing.

The InBios Scrub Typhus Detect IgM and IgG ELISAs (InBios International, Inc., Seattle, WA) were used to detect IgM and IgG antibodies against specific *O. tsutsugamushi* antigens. The laboratory used the cutoff value defined by the receiver operator characteristic curve. The method for cutoff determination is described in a pending publication: “InBios Scrub typhus IgM and IgG ELISA Cut-Off Determination for Thailand” by Teepruksa et al. (unpublished data). The cutoff value for the Scrub Typhus Detect assay was 0.579 for IgM and 1.151 for IgG. An equivocal result was defined as an optical density (OD) ± 10% of the cutoff (IgM: 0.521–0.637; IgG: 1.036–1.266). A result was considered positive if the OD was greater than the cutoff +10% (IgM: >0.637; IgG: >1.266). Results are provided as positive, equivocal, or negative. All specimens for ELISA testing were transported to the laboratory of the Bamrasnaradura Infectious Diseases Institute in Nonthaburi, Thailand. The ELISA testing results were part of a research-use-only assay evaluation and were not reported to clinicians.

#### Rapid diagnostic tests.

The rapid diagnostic tests (RDTs) were performed for *Streptococcus pneumoniae* (*S. pneumoniae*), dengue virus, and *Plasmodium* species using the same samples and diagnostic tests as specified in Wodniak et al. (2024).^24^ Rapid diagnostic tests were performed at the collecting hospital, and the results were used for clinical management.

### Case definitions and selection criteria.

Cases were classified into four groups according to the following definitions and selection criteria:

- Confirmed: Enrolled patients with a positive singleplex PCR result
- Probable: Enrolled patients who meet presumptive laboratory evidence (i.e., ELISA positive for anti-IgM/IgG *O. tsutsugamushi* antibodies) as defined below:
  ○ An acute to convalescent rise (change from negative/equivocal to positive) in anti-IgM and/or anti-IgG *O. tsutsugamushi* antibodies and at least two of the following clinical criteria^21^^,^^25^^–^^27^:
    ○ Headache
    ○ Rash
    ○ Cough or dyspnea
    ○ Myalgia
    ○ Jaundice
- Suspect: enrolled patients who meet supportive laboratory evidence (i.e., ELISA positive for anti-IgM/IgG *O. tsutsugamushi* antibodies) as defined below:
  ○ Positive acute and convalescent sera for anti-IgM/IgG *O. tsutsugamushi* antibodies or
  ○ For patients with only an acute or convalescent sera sample, positive acute or convalescent sera for anti-IgM/IgG *O. tsutsugamushi* antibodies and at least two of the clinical criteria listed above
- Non-cases: all those not classified as confirmed, probable, or suspect were considered non-cases

### Data management and statistical analysis.

Adults aged 18 to 80 years from both provinces were included in the analysis. Children were excluded from this specific analysis because of differences in epidemiologic risk factors and a too-small sample size. To ensure that the sample represented the target population of hospitalized AFI and AUFI patients, the authors assigned weights to each patient in the study. Acute febrile illness patient weights were calculated as the inverse of the proportion of sampled patients among the total number of AFI patients at that hospital. All AUFI patients were assigned a weight of 1. All analyses were performed separately for Tak and NP. Analysis accounted for the stratified sampling of individuals by AFI status (AFI or AUFI), and weighting was applied unless otherwise indicated. Design-based Clopper-Pearson confidence limits were used to calculate 95% CIs for proportions. The stability of the proportions was evaluated using National Center for Health Statistics data presentation standards.^28^ For relative risks, Wald-based 95% CIs were calculated. Data analyses were conducted using SAS 9.4 (SAS Institute, Cary, NC).

The overall prevalence estimates of confirmed ST were calculated using all participants with a singleplex PCR result. The prevalence of confirmed, probable, and suspect ST infections was also calculated from a subset of individuals with both a singleplex PCR result and IgM and/or IgG ELISA results, after excluding individuals for whom case classification status could not be determined. Prevalence estimates by demographics, clinical manifestations, and exposure history were calculated by combining confirmed and probable cases and were compared with estimates among non-cases. The clinical administration of antibiotics in the 72 hours before hemoculture was evaluated to determine if appropriate antibiotics were administered to confirmed and probable cases.

Discharge diagnoses and coinfections were described for confirmed and probable cases. The accuracy of ST discharge diagnosis was determined by comparing laboratory outcomes with the clinical diagnosis at discharge and was further evaluated in relation to possible coinfections. Coinfections were determined on the basis of positive results by either singleplex or multiplex PCR or any positive antigen RDT results. Clinical features were described for confirmed and probable cases.

## RESULTS

**Figure 1.**
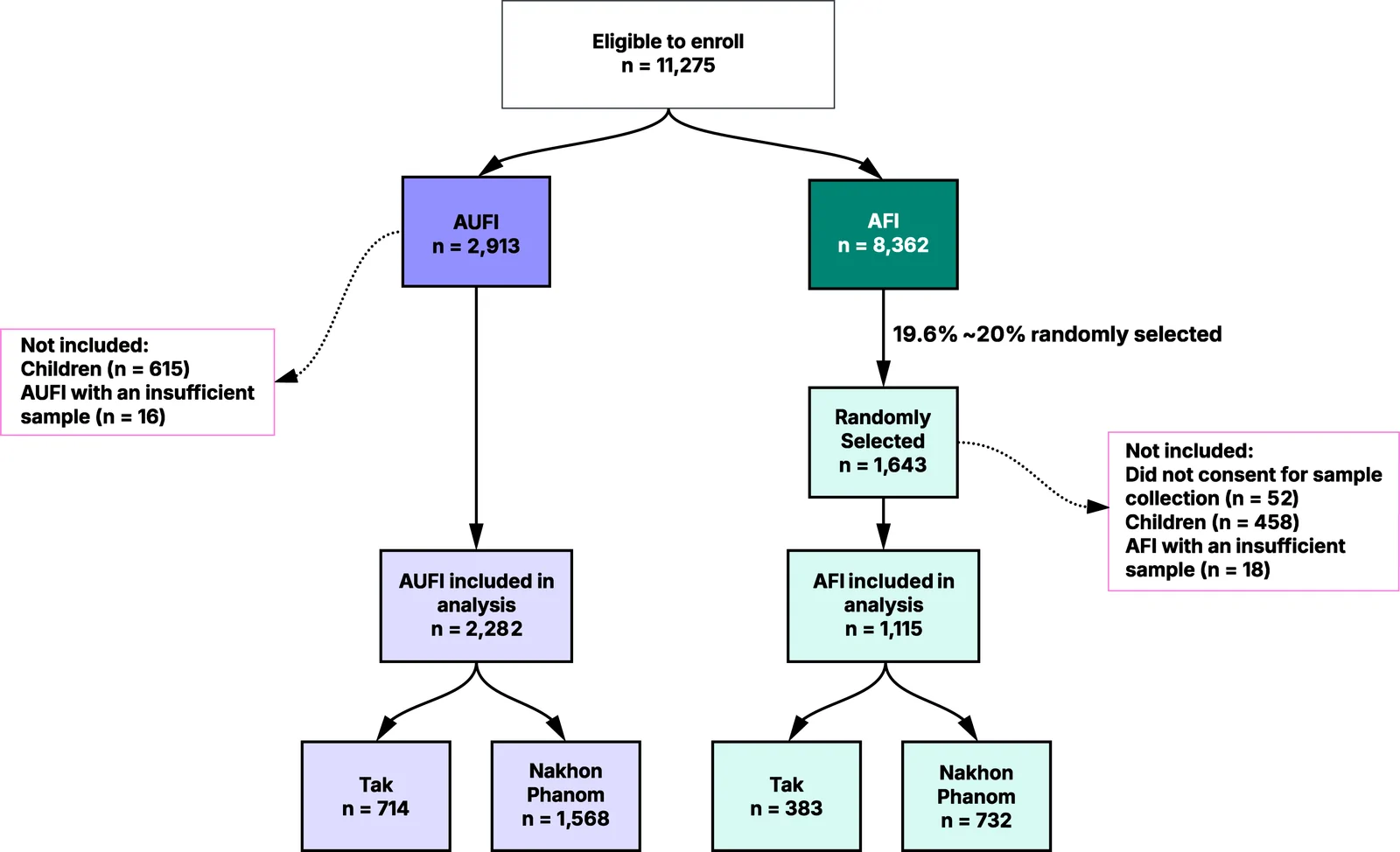
Total study participants included in scrub typhus analysis, characterized by acute febrile illness cohort and location, April 2017 to May 2020.

Of the 11,275 patients who were eligible for enrollment in the present study, 2,913 were categorized as having AUFI and consented to participate. There were 8,362 patients categorized as having AFI, of whom 1,643 were randomly selected for participation, and 1,591 consented to participate. After excluding children and patients with insufficient sample for testing from the 4,504 patients who consented to participate, 3,397 patients were included in the present analysis: 1,097 from Tak and 2,300 from NP (Figure 1).

After weighting was applied, the estimated proportion of female patients hospitalized with AUFI or AFI in Tak (59%) is similar to the proportion in NP (54%); however, patients in Tak are estimated to be younger (36% aged 18–35 years) than those in NP (17% aged 18–35 years). In Tak, 92% of patients hospitalized with AUFI or AFI are estimated as Thai ethnicity, whereas >99% are estimated as Thai ethnicity in NP. Other ethnicities included Karen, Lao, Burmese, and others, but there were too few non-Thai participants to analyze separately. Similar proportions of hospitalized AUFI or AFI patients in Tak and NP are estimated to have exposures to water (flood water, mud, puddles, ponds, rivers, creeks, or reservoirs; Tak: 30%, NP: 32%); cutting down trees, bushes, gathering wood, or clearing land (Tak: 31%, NP: 32%), and visiting the forest (Tak: 28%, NP: 25%). A smaller proportion was estimated to have had contact with rodents in Tak (16%) than those in NP (60%). Most patients are estimated to be farmers. For Tak, rice farming is estimated to be uncommon (2%), and the majority of participants are estimated to be other types of farmers (31%), whereas for NP, rice farmers (40%) make up the majority of farmers, and other types of farming are rare (3%; Table 1).

**Table 1:** Demographics, clinical manifestations, and exposure histories of adult patients with acute febrile illness, Tak and Nakhon Phanom Provinces, Thailand, April 2017 to May 2020

	Tak		Nakhon Phanom	
Characteristics	Unweighted, *n*	Weighted, %	Unweighted, *n*	Weighted, %
Overall, *N*	780	–	1,942	–
Sex				
Male	321	41.0	900	46.2
Female	459	59.0	1,042	53.8
Age (years)				
18–35	313	36.1	335	17.1
36–59	318	38.3	776	39.0
≥60	149	25.6	831	43.9
Ethnicity				
Thai	699	91.9	1,938	99.9
Other	81	8.1	4	0.1
Clinical manifestation at admission				
Fatigue	650	84.0	1,666	88.8
Headache	603	76.2	1,255	66.7
Muscle pain	580	76.1	1,358	71.9
Loss of appetite	559	71.6	1,273	66.5
Chills	565	69.1	1,333	67.5
Calf muscle pain	439	58.2	901	48.3
Back pain	429	56.6	958	50.4
Nausea	425	56.2	860	48.6
Cough	232	53.5	631	59.6
Dyspnea	209	42.2	474	41.5
Vomiting	288	39.3	631	34.9
Joint pain	299	38.4	644	35.4
Bone pain	245	33.6	400	22.0
Pale or cold skin	239	33.0	637	32.7
Chest pain	186	30.6	430	31.5
Abdominal pain	219	30.6	519	31.2
Eye pain	209	26.2	282	15.7
Dysuria	113	16.9	361	17.4
Rash	101	11.6	146	7.3
Red eyes	89	10.8	111	7.3
Yellow eyes or skin	46	6.5	132	7.2
Nose bleeding	21	3.2	26	1.5
Red or swollen joints	24	2.9	103	4.3
Stiff neck	17	2.4	85	4.5
Seizures	13	1.7	73	2.7
Bruises	12	1.1	61	3.8
Exposure history (yes)^†^				
Water (flood water, mud, puddles, ponds, rivers, creeks, or reservoirs)	251	30.3	615	31.9
Walked outside without shoes	163	22.5	717^‡^	38.2
Cut down trees, bushes, gathered wood, and/or cleared land	232	31.0	596	31.7
Visited forest	255	27.7	482	24.7
Visited or worked with rubber trees	9	1.5	310	15.7
Cut or scraped self	129^‡^	16.9	344	20.1
Farm animals (cow, buffalo, pig, goat, sheep)	198	22.6	675	34.5
Domestic birds (chicken, duck)	305	39.7	1197^‡^	62.5
Dog	359	49.8	1189	61.6
Cat	230^‡^	28.9	440	22.2
Rat or rodent	126^§^	16.1	1,186^§^	60.1
Stray animals	45	7.4	297	14.5
Bitten by an insect other than a mosquito	132^‡^	16.0	572^‖^	28.4
Went abroad	60	8.3	17	1.0
Occupation				
Rice farmer	14	1.9	766	40.2
Fish farmer	0	0	0	0
Farmer (other)	257	31.0	61	3.2
Fisherman	5	0.30	2	0.04
Rancher	0	0.00	3	0.30
Forest worker/gardener	1	0.06	3	0.50
Doctor	2	0.12	1	0.10
Nurse	11	1.1	8	0.50
Government worker	46	4.2	81	4.1
Ranger	0	0	1	0.10
Monk	2	0.36	14	0.80
Housekeeper	51	6.9	67	2.9
Unemployed	99	16.3	473	23.8
Student	25	3.0	41	2.3
Retired	20	3.2	49	2.4
Preschool children	0	0	0	0
Merchant	51	8.0	125	6.5
Casual laborer	139	15.7	183	8.8
Other	57	7.9	64	3.4
Indoor (doctor, nurse, government worker, monk, housekeeper, merchant)	163	20.8	296	15.0
Outdoor (rice farmer, fish farmer, farmer [other], fisherman, rancher, forest worker/gardener, ranger, casual laborer)	416	48.9	1,019	53.1
Other (unemployed, student, retired, preschool children, other)	201	30.3	627	31.9
Water work (rice farmer, fisherman, fish farmer)	19	2.2	768	40.2

* Confirmed and probable scrub typhus cases were combined and used to determine prevalence.

^†^ Within the 30 days before hospital admission.

^‡^ Response missing for one person.

^§^ Response missing for three people.

^‖^ Response missing for two people.

**Figure 2.**
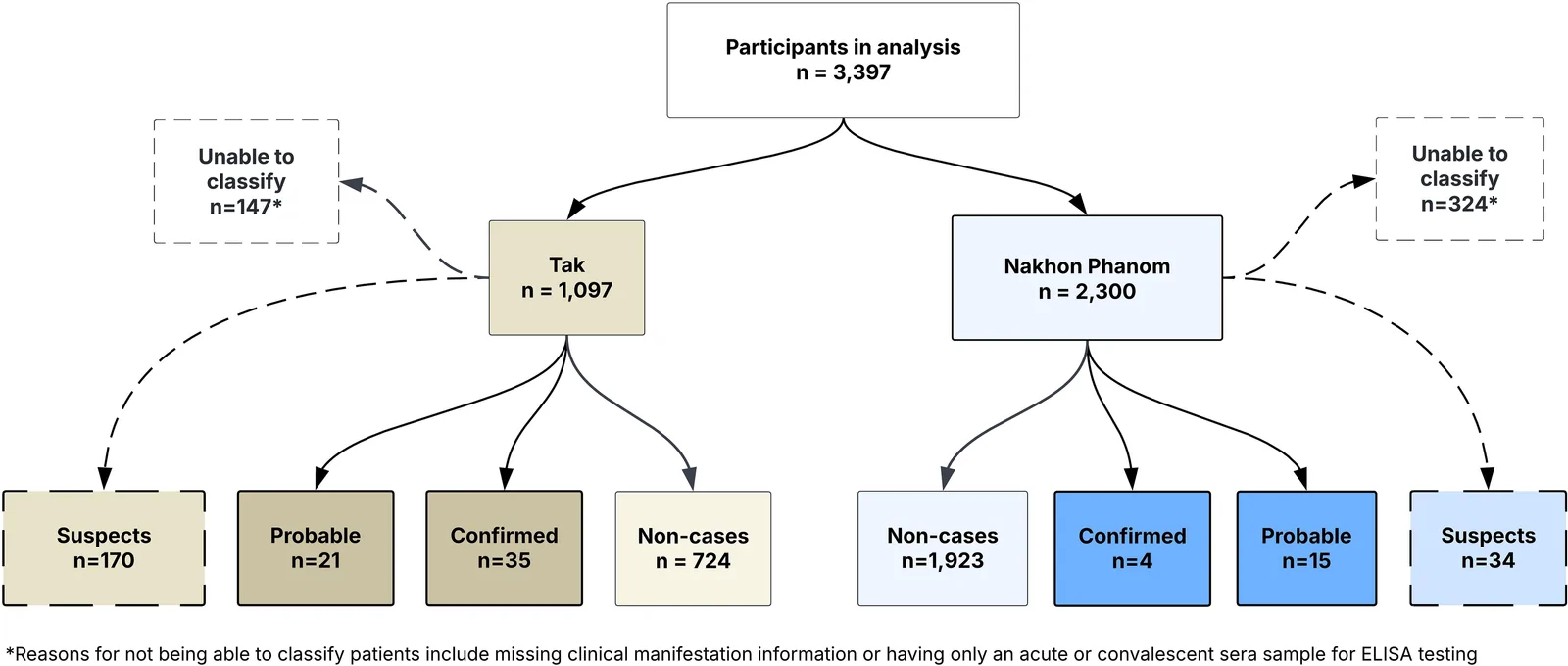
Study participants categorized by location and scrub typhus case definition designation, April 2017–May 2020.

For Tak Province, 35 patients were classified as confirmed ST cases, 21 were classified as probable, 724 were classified as non-cases, 170 were classified as suspect, and 147 were unable to be classified, leaving 780 adults in the analysis. For NP Province, four patients were classified as confirmed ST cases, 15 were classified as probable, 1,923 were classified as non-cases, 34 were classified as suspect, and 324 were unable to be classified, leaving 1,942 adults in the analysis (Figure 2).

Laboratory-confirmed coinfection was noted in 18 (24%) of the 75 combined confirmed and probable cases in Tak and NP. Of the 39 confirmed ST cases in Tak and NP, one in Tak was coinfected with *E. coli*. Of the 36 probable ST cases in Tak and NP, three in Tak were coinfected with chikungunya, five in Tak and six in NP were coinfected with *Leptospira*, one in NP was coinfected with *B. pseudomallei*, and two in Tak were coinfected with *S. pneumoniae*.

The overall estimated prevalence of ST in the hospitalized adult population in Tak is 6.9%; this estimate is higher than the 1.3% estimate for NP (Tables 2 and 3). In both Tak and NP, differences in prevalence were not statistically significant between males and females or between age groups.

**Table 2:** Prevalence of scrub typhus in adults hospitalized with acute febrile illness, Tak and Nakhon Phanom Provinces, Thailand, April 2017 to May 2020

	Tak			Nakhon Phanom		
Characteristics	Unweighted, *n*	Prevalence of Scrub Typhus* (95% CI)	RR (95% CI)	Unweighted, *n*	Prevalence of Scrub Typhus* (95% CI)	RR (95% CI)
Overall, *N*	780	6.89 (4.84–9.42)	–	1,942	1.26 (0.62–2.25)	–
Sex						
Male	321	5.98 (3.22–10.02)	Ref.	900	1.97 (0.82–3.94)	Ref.
Female	459	7.50 (4.78–11.09)	1.25 (0.64–2.44)	1,042	0.64 (0.15–1.78)	0.33 (0.09–1.18)
Age (years)						
18–35	313	5.88 (3.04–10.12)	Ref.	335	2.52 (0.75–6.09)	Ref.
36–59	318	10.58 (6.56–15.91)	1.79 (0.89–3.62)	776	1.21 (0.4–2.77)	0.48 (0.13–1.71)
≥60	149	^†^	–	831	0.81 (0.1–2.85)	0.32 (0.06–1.68)
Ethnicity						
Thai	699	6.12 (4.14–8.66)	–	1,938	1.26 (0.62–2.26)	–
Other	79	^†^	–	4	^†^	–

Ref. = reference.

* Confirmed and probable scrub typhus cases were combined and used to determine prevalence; the suspect cases and those unable to be confirmed have been removed from the denominator. Prevalence estimates are after weighting has been applied.

^†^ These estimates are unstable, so they have been suppressed.

**Table 3:** Proportion of participants by case classification in adults hospitalized with acute febrile illness, Tak and Nakhon Phanom Provinces, Thailand, April 2017 to May 2020

	Tak		Nakhon Phanom	
Case Classification	Unweighted, *n*	Weighted Proportion in Each Category (95% CI)	Unweighted, *n*	Weighted Proportion in Each Category (95% CI)
Confirmed	1,095*	2.89 (1.82–4.33)	2,298*	0.07 (0.01–0.28)
Confirmed	950^†^	3.33 (2.10–4.99)	1,976^†^	0.08 (0.01–0.33)
Probable	950^†^	2.13 (1.15–3.61)	1,976^†^	1.15 (0.54–2.14)
Suspect	950^†^	20.54 (17.30–24.08)	1,976^†^	2.24 (1.36–3.46)
Non-cases	950^†^	74.00 (70.25–77.51)	1,976^†^	96.54 (95.09–97.65)

* *N* includes all participants with polymerase chain reaction results and incomplete data.

^†^ *N* excludes those whose case classification could not be determined. This *N* also differs from the analytic sample, which further excludes suspect cases.

### Clinical features.

In Tak, the three most common symptoms among those with confirmed or probable ST (*n* = 56) were headache (*n* = 51; 92%), fatigue (*n* = 49; 87%), and chills (*n* = 50; 87%). In contrast, for the overall Tak study population (*N* = 780), the most common symptoms were fatigue (*n* = 650; 84%), headache (*n* = 603; 76%), and myalgia (*n* = 580; 76%). In NP, the three most common symptoms among confirmed and probable ST (*n* = 19), in addition to fever, were headache (*n* = 19; 100%), fatigue (*n* = 17; 97%), and myalgia (*n* = 16; 88%), whereas for the NP overall study population (*N* = 1,942) they were fatigue (*n* = 1,666; 89%), myalgia (*n* = 1,358; 72%), and chills (*n* = 1,325; 68%).

### Accuracy of discharge diagnosis.

Of the 56 confirmed and probable ST cases in Tak, 10 (18%) patients were discharged with diagnoses of ST, and four (7%) were discharged with diagnoses of rickettsiosis. In NP, one (5%) patient of 19 confirmed and probable ST cases received a discharge diagnosis of ST. Of the 56 confirmed or probable ST cases in Tak, three (5%) patients had laboratory-confirmed coinfections, all of whom had a correct corresponding discharge diagnosis of chikungunya; of the 19 cases in NP, three (16%) patients had laboratory-confirmed coinfections, two of whom had a matching corresponding discharge diagnosis of leptospirosis, and one of whom was diagnosed with melioidosis. None of the 36 probable cases in either province was diagnosed as ST at discharge. For Tak, 11 (20%) of the 56 confirmed and probable ST cases were discharged with a diagnosis of dengue, all of whom tested negative for dengue via PCR or antigen RDT (Supplemental Table 1).

### Antibiotic therapy within 72 hours before hemoculture.

Of the 75 confirmed and probable ST cases, 16 (21%) patients received antibiotics within 72 hours before hemoculture collection, with six (40%) receiving more than one type of antibiotic. Of those 16, nine (56%) received an antibiotic (doxycycline) appropriate for ST (Supplemental Table 2). Second- (cefuroxime) and third-generation (ceftriaxone and cefotaxime) cephalosporins were administered to 12 (75%) patients, and metronidazole was administered to one (6%) patient.

### Exposure history.

Various environmental and animal exposures were assessed to determine associations with ST infection. Hospitalized patients with AUFI or AFI in Tak were 4.2 (95% CI: 2.2–8.2) and 4.5 (95% CI: 2.4–8.5) times as likely to test positive for ST if they visited the forest or contacted farm animals (e.g., cows, buffaloes, pigs, goats, sheep), respectively, within the past 30 days. The following activities were associated with a higher risk of infection: contact with water (RR: 2.1; 95% CI: 1.1–3.9); cutting down trees, gathering wood, or clearing land (RR: 2.0; 95% CI: 1.1–3.8); and exposure to rodents (RR: 2.1; 95% CI: 1.1–4.2). Those with insect bites other than from mosquitoes also had a higher risk of infection (RR: 2.6; 95% CI: 1.3–5.0). In contrast, there was not a statistically significant association for contact with dogs (RR: 1.4; 95% CI: 0.7–2.6) or cats (RR: 1.8; 95% CI: 0.9–3.4; Table 4). For NP, none of the assessed exposures were statistically significantly associated with ST infection, except visiting the forest (RR: 4.9; 95% CI: 1.5–16.2). For Tak, 49% of hospitalized patients with AUFI or AFI reported outdoor occupations, and in NP, the figure was 53%. The risk of ST in people who work outdoors in Tak is 20 times that of those who do not work outdoors, whereas in NP, that risk is 2.6 times higher.

**Table 4:** Exposure variables for scrub typhus risk factors, adults in Tak and Nakhon Phanom Provinces, Thailand, April 2017 to May 2020

		Tak (*N* = 780)							Nakhon Phanom (*N* = 1,942)					
		Exposed			Unexposed				Exposed			Unexposed		
Exposure History*	Scrub Typhus (+), *n*	Exposed, *n*	Prevalence^†^	Scrub Typhus (+), *n*	Exposed, *n*	Prevalence^†^	RR (95% CI)	Scrub Typhus (+), *n*	Exposed, *n*	Prevalence^†^	Scrub Typhus (+), *n*	Exposed, *n*	Prevalence^†^	RR (95% CI)
Overall														
Water (flood water, mud, puddles, ponds, rivers, creeks, or reservoirs)	28	223	10.7%	28	501	5.2%	2.1 (1.1–3.9)	13	615	2.2%	6	1,327	0.8%	2.6 (0.8–5.6)
Walked outside without shoes	19	144	10.2%	37	580	5.9%	1.7 (0.9–3.4)	9	717	1.0%	10	1,224	1.4%	0.7 (0.2–2.3)
Cut down trees, bushes, gathered wood, and/or cleared land	27	205	10.5%	29	519	5.3%	2.0 (1.0–3.8)	5	596	1.7%	14	1,346	1.1%	1.6 (0.4–5.5)
Visited forest	36	219	15.4%	20	505	3.6%	4.2 (2.2–8.2)	13	482	3.1%	6	1,460	0.6%	4.9 (1.5–16.2)
Farm animals (cow, buffalo, pig, goat, sheep)	32	166	17.3%	24	558	3.8%	4.5 (2.4–8.5)	11	675	1.5%	8	1,267	1.1%	1.3 (0.4–4.5)
Domestic birds (chicken, duck)	38	267	11.9%	18	457	3.5%	3.4 (1.7–6.8)	5	1,197	1.6%	19	744	0.7%	2.1 (0.6–7.8)
Dog	30	329	8.0%	26	395	5.8%	1.4 (0.7–2.6)	13	1,189	1.3%	6	753	1.2%	1.0 (0.3–3.3)
Cat	27	203	10.0%	29	520	5.6%	1.8 (0.9–3.3)	5	440	0.5%	14	1,502	1.5%	0.3 (0.1–0.9)
Rat or rodent	18	108	12.3%	38	613	5.9%	2.1 (1.0–4.2)	14	1,186	1.6%	5	753	0.7%	2.3 (0.6–8.6)
Bitten by an insect other than a mosquito	18	114	14.1%	38	609	5.5%	2.6 (1.3–5.0)	9	572	1.4%	10	1,368	1.2%	1.2 (0.4–4.0)
Occupation														
Rice farmer	7	14	^‡^	–	–	–	–	9	766	1.2	–	–	–	–
Fish Farmer	0	0	0	–	–	–	–	0	0	0	–	–	–	–
Farmer (other)	37	257	14.7	–	–	–	–	2	61	^‡^	–	–	–	–
Fisherman	0	5	0	–	–	–	–	0	2	0	–	–	–	–
Rancher	0	0	0	–	–	–	–	0	3	0	–	–	–	–
Forest worker/gardener	0	1	0	–	–	–	–	0	3	0	–	–	–	–
Doctor	0	2	0	–	–	–	–	0	1	0	–	–	–	–
Nurse	0	11	0	–	–	–	–	0	8	0	–	–	–	–
Government worker	1	46	^‡^	–	–	–	–	0	81	0	–	–	–	–
Ranger	0	0	0	–	–	–	–	0	1	0	–	–	–	–
Monk	0	2	0	–	–	–	–	0	14	0	–	–	–	–
Housekeeper	1	51	^‡^	–	–	–	–	1	67	^‡^	–	–	–	–
Unemployed	4	99	^‡^	–	–	–	–	2	473	0.7	–	–	–	–
Student	2	25	^‡^	–	–	–	–	0	41	0	–	–	–	–
Retired	1	20	^‡^	–	–	–	–	0	49	0	–	–	–	–
Merchant	0	51	0	–	–	–	–	0	125	0	–	–	–	–
Casual laborer	3	139	^‡^	–	–	–	–	5	183	^‡^	–	–	–	–
Other	0	57	0	–	–	–	–	0	64	0	–	–	–	–
Occupation groups														
Indoor (doctor, nurse, government worker, monk, housekeeper, merchant)	2	163	0.6	–	–	–	Ref.	1	296	0.7	–	–	–	Ref.
Outdoor (rice farmer, fish farmer, farmer (other), fisherman, rancher, forest worker/gardener, ranger, casual laborer)	47	416	11.5	–	–	–	19.8 (4.7–83.3)	16	1,019	1.9	–	–	–	2.6 (0.3–20.9)
Other (unemployed, student, retired, preschool children, other)	7	201	^‡^	–	–	–	^‡^	2	627	0.4	–	–	–	0.6 (0.0–7.7)

Ref. = reference.

* Within 30 days before hospital admission.

^†^ These estimates are weighted.

^‡^ These estimates are unstable, so they have been suppressed.

## DISCUSSION

The present study highlights a persistent and geographically variable burden of ST along two of Thailand’s border provinces, with higher detection in the north.^29^ These findings align with the earlier literature indicating that ST remains an important cause of AFI in rural and border areas with limited diagnostic and surveillance capacities.^19^^,^^29^^–^^32^ The concentration of cases in Tak, which has been reported as having Thailand’s fourth highest ST incidence rate in 2019 (63.77 per 100,000 population),^29^ likely reflects ecological and occupational risk factors, including dense forest cover (72% of the province area), favorable habitats for mites, and greater human exposure through agricultural and forest-related activities.^19^^,^^32^^–^^34^ In contrast, NP, with only 14% forest cover, exhibited a lower prevalence, consistent with previous data, indicating a low prevalence of ST disease in NP.^29^ This also mirrors ST seroprevalence estimates of 48% among the hill tribes in Northern Thailand versus 4.2% within the Northeastern Region.^4^^,^^19^^,^^35^

Despite official Epidemiologic Surveillance Reports (R506) that specify zero ST cases in NP province in 2019,^29^ the study data revealed five patients (one confirmed ST and four probable ST; none had a discharge diagnosis of ST) who were hospitalized in NP in the same year. The Thailand Bureau of Epidemiology monitors ST through the national R506, in which cases are classified as suspect (fever or eschar with compatible clinical symptoms [e.g., headache, myalgia, red eyes, cough, rash, lymphadenopathy, jaundice, pneumonia]), probable (suspected case criteria with presumptive diagnosis), and confirmed (suspected case criteria with specific diagnostics such as PCR or serology testing). Laboratory diagnostics for ST in rural hospitals are not routinely available, which can further contribute to underreporting of ST cases.

Risk factors for ST infections in Thailand include contact with certain domestic animals, vegetation, and damp land,^35^ as well as sanitation, occupation, and socioeconomic factors.^19^^,^^36^^,^^37^ In the present analysis, 23% of participants from Tak and 35% of participants from NP reported contact with farm animals, but the differing ST prevalence between provinces suggests that animal contact alone may not be the primary exposure risk. It may instead indicate farm animals being housed in places that are better ST mite habitats in Tak. Alternatively, the prevalence of ST in NP and the sample size from NP are too low to draw conclusions.

The two provinces have similar proportions of participants reporting outdoor occupations. The increased risk for ST in those working outdoors is consistent with prior publications.^19^

For Tak, reporting visiting the forest; contact with flood water, mud, puddles, ponds, rivers, creeks, or reservoirs (i.e., contact with water); cutting down trees or bushes, gathering wood, or clearing land; contact with domestic birds; and contact with rodents may serve as proxies for exposure to ST-infected mites, as noted in other studies.^36^^,^^38^ However, for NP, the association between risk and these exposures, other than visiting the forest, is weaker, which may be due to the lower prevalence of ST in this province or insufficient numbers of participants from NP to draw conclusions about exposure risks for ST. Standardized protocols for collecting detailed exposure histories in patients hospitalized with AFI in these provinces should be routinely implemented, with the abovementioned environmental exposures considered for inclusion. The use of personal protective equipment when working in fields was found to be a protective factor in other studies.^35^^,^^36^

Compared with previous epidemiological studies conducted in Thailand, in which immunofluorescent antibody (IFA) results have been used as part of the ST case definition,^21^^,^^30^ the present investigation adds to the understanding of ST distribution and epidemiology through the use of diagnostic data from ELISA. Whereas IFA interpretation requires skill and can be subjective, ELISA involves the use of optical densities, which have objective interpretations. However, determining ELISA OD cutoffs is complex and population-specific; validating these cutoffs and assessing OD changes over time would improve diagnostic reliability. Currently, the ELISA test used is only available for research purposes; this test might be a more accessible tool in low-resource settings if it is approved for diagnostic use.

Early detection and timely treatment of ST remain challenging because of its nonspecific clinical manifestations. The clinical presentation observed in confirmed and probable ST cases here, specifically headache, myalgia, chills, and fatigue, is consistent with previous publications.^9^^,^^25^^,^^39^ Although ST and Rickettsiosis share similar clinical features and treatment, only 25% (14 of 56) of confirmed or probable ST cases in Tak and NP were discharged with either diagnosis. This may be due to an unreliable case definition, a lack of point-of-care testing, and challenges in clinical recognition. The authors of a study conducted in Vietnam proposed a clinical algorithm for use in low-resource settings to differentiate dengue and ST in endemic areas^9^; applying it in Thailand could improve case detection. Misclassification with other febrile illness etiologies, such as dengue, as observed in ST cases in Tak, underscores the need for improved low-cost point-of-care diagnostic tools to support accurate identification and ensure timely and appropriate treatment of ST and other related infections in endemic areas.^20^ Coinfections of ST with malaria,^30^ leptospirosis,^20^^,^^40^ other rickettsioses,^41^ or dengue^20^ have been previously described and contribute to the difficulty diagnosing and treating febrile illnesses. In the present analysis, the three confirmed or probable ST cases coinfected with chikungunya were accurately diagnosed, two of 15 probable ST cases coinfected with leptospirosis and one of 15 probable ST cases coinfected with melioidosis were accurately diagnosed with these diseases, but none of these patients were diagnosed with the additional coinfection of ST.

Inappropriate use of antibiotics is another concern; 44% (7/16) of participants received ceftriaxone, a third-generation cephalosporin, without concurrent use of an antibiotic effective against ST. A study conducted in Thailand’s Chiang Rai province revealed that 17.6% of ST and rickettsial disease patients received antibiotics that were ineffective for the disease.^22^ However, the present study’s data cannot be compared directly because the authors only have information from medical records about antibiotic use before hemoculture. Continued surveillance of ST cases and antibiotic usage patterns in the community can help identify trends, inform public health strategies, and guide future research on effective interventions in Thailand and across its borders.

To improve the detection and management of ST in Thailand’s border regions, several actions are recommended. First, awareness among clinicians and health workers should be strengthened through targeted training on the clinical features of and risk factors for ST, particularly in endemic areas. This can support more accurate syndromic recognition and prompt empiric treatment, especially where diagnostic capacity is lacking. Second, a clinical algorithm for differentiating ST from other AFI diseases should be validated for use in Thailand to assist with clinical decision-making. Third, an antigen-based low-cost point-of-care ST diagnostic test should be developed for use in resource-limited settings. A reliable, low-cost point-of-care diagnostic test could greatly improve clinicians’ ability to identify ST and prescribe appropriate antibiotic therapy, thereby improving antimicrobial stewardship and improving patient outcomes.^22^^,^^42^ However, in some areas, doxycycline, the first-line medication for ST, may be intermittently unavailable, which can delay appropriate treatment. Thus, improving both diagnostic access and treatment availability is essential in areas with high ST prevalence. Finally, surveillance systems should be enhanced by standardizing specimen collection and history-taking practices at the local level. Strengthened laboratory networks and data-sharing mechanisms would allow for more timely outbreak detection and response.

### Limitations.

The present study has several limitations. First, the study results are not representative of the whole country because only hospitalized individuals were included; the study included only two rural Thai border provinces, which vary greatly in ST prevalence, and included only legal residents of Thailand. Scrub typhus can often cause mild symptoms that do not require hospitalization; thus, prevalence estimates are also likely underestimated. Second, the case report form used for the AFI study was a general one designed to collect information on multiple AFI pathogens; thus, the authors do not have information on the presence of eschars for patients, which can be a helpful diagnostic clue for ST diagnosis when present.^10^^,^^25^ Third, information about participants’ living environment was not collected as part of the AFI study from which the present study data were obtained, but they may be helpful to include in patient history when attempting to diagnose an AFI. Fourth, the number of confirmed or probable ST cases is low in this population sample, which contributes to wide CIs and unstable estimates for RRs and prevalence in this analysis. Fifth, the authors were unable to use laboratory values such as platelet counts, alanine aminotransferase, and aspartate aminotransferase as case definition criteria because of high levels of missing information. These laboratory values were available only if clinicians ordered these tests within 24 hours of admission; therefore, the authors determined that they would not be included in the case definition for this analysis. Finally, information about antibiotic use was limited to 72 hours before hemoculture; thus, the authors did not have information about treatment during the participants’ full hospitalization or at discharge.

## CONCLUSION

Scrub typhus remains a noteworthy and underrecognized cause of AFI in two of Thailand’s northern border provinces. The present study provides updated information on the clinical, epidemiological, and laboratory characteristics of ST across different geographic areas of Thailand. It highlights continued gaps in the detection, diagnosis, and surveillance of ST in rural and resource-limited settings. Strengthening clinical awareness and laboratory capacity are essential to improve early identification, case management, and public health response. Sustained investment in these areas will be key to reducing the disease burden and preventing outbreaks in vulnerable border populations.

## Supplemental Materials

10.4269/ajtmh.25-0551 Supplemental Materials
