# Supplementary material for: Clinical and Laboratory Features of Scrub Typhus among Adult Patients with Acute Febrile Illness in Tak and Nakhon Phanom Provinces, Thailand, 2017–2020
Source: Am J Trop Med Hyg. 2026 Jul 14;115(3):453–63. doi: 10.4269/ajtmh.25-0551 (PMC13539749; doi:10.4269/ajtmh.25-0551)
Supplement: Supplementary file 1 [file tpmd250551.SD1.pdf]

Supplement Table 1. Discharge Diagnoses of Adult Participants by Case Classification, Tak and Nakhon Phanom provinces, Thailand, April 2017-May 2020.

	**Tak**		**Nakhon Phanom**	
**Discharge Diagnosis**	Confirmed (n = 35)	Probable (n = 21)	Confirmed (n = 4)	Probable (n = 15)
Chikungunya		3		
Dengue	10	1		1
Influenza				1
Leptospirosis				2
Melioidosis				1
Other*	12	14	3	10
Rickettsiosis	3	1		
**Scrub Typhus**	**10**		**1**	
Tuberculosis		1		
Varicella		1		

*Other includes: acute pyelonephritis, acute respiratory failure, ameba, bacterial meningitis, fever of unknown origin, gastroenteritis, hepatitis, pneumonia, urinary tract infection

Supplement Table 2. Antibiotic use within the 72 hours prior to hemoculture sample collection in adults with confirmed or probable scrub typhus, Tak and Nakhon Phanom provinces, Thailand, April 2017-May 2020.

	Tak		Nakhon Phanom	
Scrub typhus categorization:	Confirmed (n = 35)	Probable (n = 21)	Confirmed (n = 4)	Probable (n = 15)
Antibiotic use 72 hours prior to sample collection?				
Yes	13	3	0	0
No	21	18	4	15
No Hemoculture	1	0	0	0
Antibiotics used*:				
Doxycycline	4			
Ceftriaxone	4	2		
Ceftriaxone and doxycycline	2	1		
Ceftriaxone and metronidazole	1			
Cefotaxime and doxycycline	1			
Cefuroxime and doxycycline	1			

*Antibiotic use information came from participant's medical charts. Antibiotics with effectiveness against scrub typhus include: doxycycline, chloramphenicol, and azithromycin.
